# Fluorescence-aided removal of orthodontic composites: an in vivo comparative study

**DOI:** 10.1186/s40510-022-00411-w

**Published:** 2022-05-23

**Authors:** Paolo Albertini, Rosita Tauro, Lorenza Barbara, Enrico Albertini, Luca Lombardo

**Affiliations:** Postgraduate School of Orthodontics, University of Ferrara, Via Livatino, 9, 42124 Reggio Emilia, Italy

**Keywords:** Fluorescence-aided identification technique, Bracket debonding, Composite resin detection, Adhesive removal

## Abstract

**Background:**

To compare the fluorescent properties of 6 different orthodontic adhesives and provide useful information for clinicians in the adhesion choice, in order to remove it easily at the end of orthodontic treatment by using the Fluorescence-aided Identification Technique (FIT).

**Methods:**

Six orthodontic adhesives were included: Ortho Connect, Gradia LoFlo A3.5, Greengloo, Transbond XT, KommonBase Pink, and KommonBase Clear. The same thermoformed template with 1 mm shell thickness on the six anterior teeth was used for adhesive positioning; furthermore, an ultraviolet light-emitting diode flashlight was used for the FIT. The brightness of adhesive area and tooth area (L* color coordinate) were measured on the photographs by using the “color picker” tool of Photoshop software.

**Results:**

GC Ortho Connect, Gradia Direct LoFlo and KommonBase Clear showed the highest differences of brightness (15.5, 16.3 and 13.5, respectively), while Greengloo, Transbond XT and KommonBase Pink registered similar values between resin area and tooth area with FIT (− 0.5, − 0.8 and − 1.0, respectively). The high viscosity adhesive resins, as Greengloo and Transbond XT, showed a similar performance in terms of fluorescence to the KommonBase Pink, the lowest viscous resin adhesive considered.

**Conclusions:**

The most used orthodontic adhesives showed different fluorescence properties. Some resins were brighter with the FIT, facilitating identification and subsequent removal. Other orthodontic adhesives presented no difference between adhesive and tooth. The viscosity of orthodontic adhesives did not influence the brightness emitted with FIT.

## Introduction

The orthodontic debonding procedure requires particular attention in removing excess bonding remnants in order to prevent dental plaque accumulation, decalcification, and carious lesions, while minimizing enamel damages [1–4]. According to a systematic review, there is no consensus regarding the most efficient technique to remove adhesive remnants [5]; the composite removers, as tungsten carbide burs, polishers and disks, could be used; however the risk of enamel loss and roughening still exists [1, 5].

Fluorescence-aided Identification Technique (FIT) is a useful method to differentiate resin composites from tooth substance by using an illumination source emitting blue light. Differences in terms of fluorescent properties between composite materials and dental hard tissues at wavelengths range of 405 ± 10 nm are used [6–9]. FIT represents the more accurate, reliable, non-invasive, and time-saving method to correctly remove adhesive [2, 8]. It facilitates the removal of composite bonded splints and detection of tooth-colored composite in restored teeth [10–12]. Furthermore, several studies have reported that auxiliary devices are useful for removal of fluorescent residue after bracket debonding, allowing the selective removal of adhesive and preserving the dental tissue [13–15]. Thus, fluorescence-aided composite removal can be used in orthodontics not only during lingual and buccal bracket debonding, but also during the removal of attachments in clear aligner therapy [16, 17].

The esthetic outcome of composites was improved with fluorescent materials, such as rare earths oxides (e.g., europium, cerium and ytterbium), which are included in glass fillers of resin for emulating the fluorescence behavior of the natural tooth [6]. However, the precise composition and concentration of these fluorescent additives are often not declared by manufacturers and are still unknown [8]. As a result, the fluorescence of adhesive resins may vary. As reported previously differences in fluorescent properties of resin composites can be found not only between same shades of different brands, but discrepancies in the fluorescence spectrum intensities can also be detected between different shades of the same brand [8].

### Aim

The fluorescence behavior of composite resins was investigated in the restorative dentistry field [6, 7, 18]; however, the orthodontics materials have not been analyzed yet. For this reason, in order to give a clinical guide in the adhesive choice for easy removal, the aim of this study was to compare the fluorescent properties of 6 different orthodontic adhesives.

## Methods

This in vivo study provides useful information for clinicians to remove bonding remnants at the end of orthodontic treatment by using the Fluorescence-aided Identification Technique (FIT).

The test patient was 26 years old, showed a full natural permanent dentition, no restorative treatment, brachyfacial skeletal pattern, slight anterior crowding, molar and canine Class I. The upper frontal teeth, from upper right canine to the left canine were considered. Frontal teeth were preferred over posterior ones to simplify photographic reliability.

In the present study, 6 orthodontic adhesives were included: Ortho Connect (GC America, Alsip, IL, USA), Gradia LoFlo A3.5 (GC America, Alsip, IL, USA), Greengloo (Ormco, Glendora, Calif, USA), Transbond XT (3M Unitek, Monrovia, CA, USA), KommonBase Pink (GC America, Alsip, IL, USA) and KommonBase Clear (GC America, Alsip, IL, USA).

**Fig. 1:**
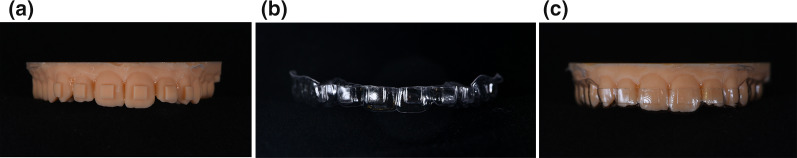
**a** Upper arch model with same adhesive thickness; **b** resin template; **c** template positioning on the model

The upper arch scan was taken using an intraoral scanner (CS 3600; Carestream Dental, Stuttgart, Germany) in order to obtain a standardized template (Erkolen 0.8 mm, ERKODENT Erich Kopp GmbH, Pfalzgrafenweiler, Germany) with the same adhesive thickness for each measurement (Fig. 1a–c).

The shells for adhesives area were designed with 3Shape Appliance Designer (3shape, Copenhagen, Denmark).

The.stl file was imported into the software, an area (3.5 × 3.5 mm) was measured with the digital ruler in the center of each tooth from upper right canine to the left canine and a 1 mm shell thickness was created for each area.

The standardized template was thermoformed on the shells models and it was cut on the top of the shells, in order to be easily removed from the arch.

The adhesives were positioned within the template shells, which were placed on the upper arch of the test patient and photo-curing was done from both top and bottom sides via 40 s-irradiation with a light-curing unit (VALO® light-curing unit, Ultradent Products GmbH, Köln, Germany). The etching and bonding procedures were avoided, in order to facilitate the subsequent removal of the material without alterations of surface roughness.

The procedure was repeated six times, each time with a different adhesive. Every time the same amount of the tested adhesive has been inserted into the shell.

According to previous studies, changes in fluorescence properties of resin adhesives may occur after aging; for this reason, all adhesives were stored according to the manufacturer's recommendation [6, 7, 19–21].

This study describes the fluorescence-aided adhesive removal with an ultraviolet (UV; 395 nm wavelength) light-emitting diode (LED) Veetop (Indialantic, Florida) flashlight, already used in a previous article [16].

The fluorescence emitted was evaluated with digital photographs obtained by a digital camera (Nikon D750, Nikon Corporation, Tokyo, Japan) equipped with Micro-Nikkor 105 mm (AF-S VR Micro-Nikkor 105 mm f/2.8G IF-ED, Nikon Corporation, Tokyo, Japan). All photographs were taken with standardized parameters: 1/60 shutter speed, f16, ISO 5000, without flash.

The camera was stabilized with a floor tripod (Manfrotto, 190 aluminum 3-section tripod with head, © 1996–2019 Vitec Imaging Solutions Spa, Cassola, Vicenza, Italy) in a standard position, frontal to the upper teeth of the test patient; the UV LED flashlight was stabilized with a flexible arm (Manfrotto, flexible arm, smartphone clamp, 035 super clamp, © 1996–2019 Vitec Imaging Solutions Spa, Cassola, Vicenza, Italy) directly orientated on the upper frontal teeth surfaces, in order to simulate the flashlight position during orthodontic debonding.

**Fig. 2:**
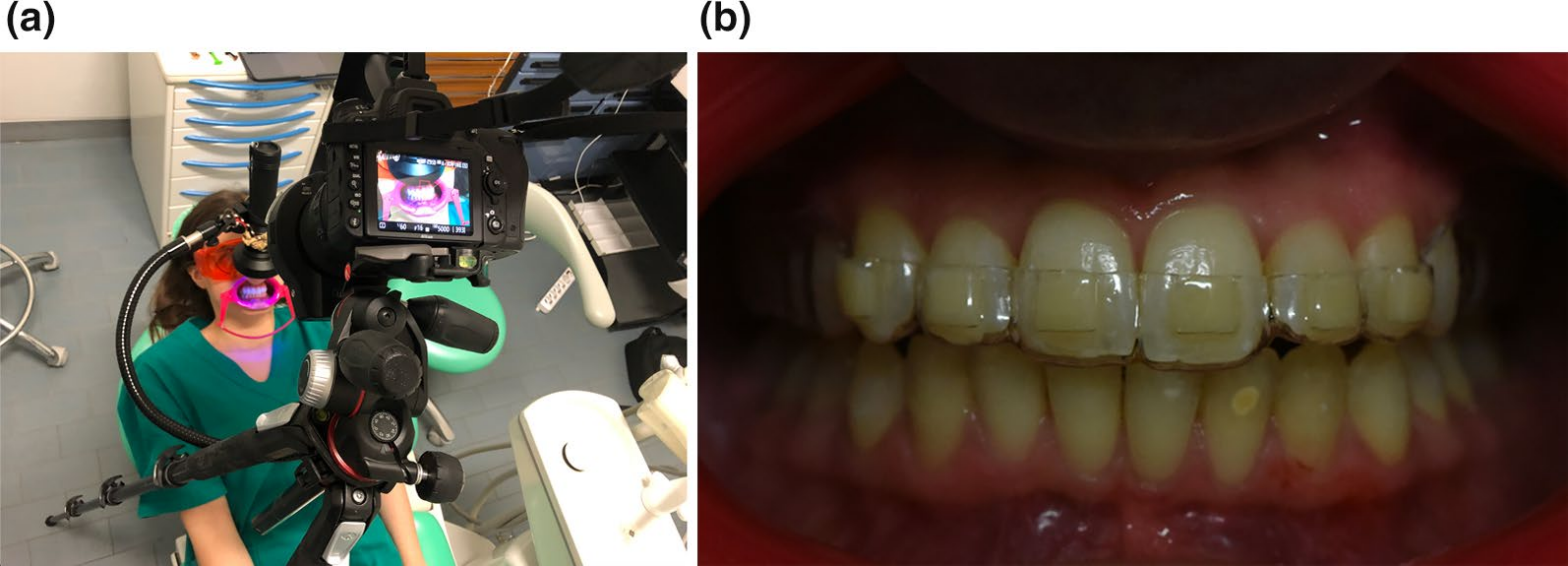
**a** Patient positioning; **b** clinical template placement

The head of the patient was maintained in the same position with a comfortable headrest, the eyes were protected with UV glasses and the cheeks were retracted with the external part of Nola Dry Field System (C-Type Cheek Retractor, Sino Dental Group, China) (Fig. 2a, b).

Adhesives were placed and photographs were taken consecutively under standardized conditions on the same day in order to maintain the same room light of the surrounding environment.

**Fig. 3:**
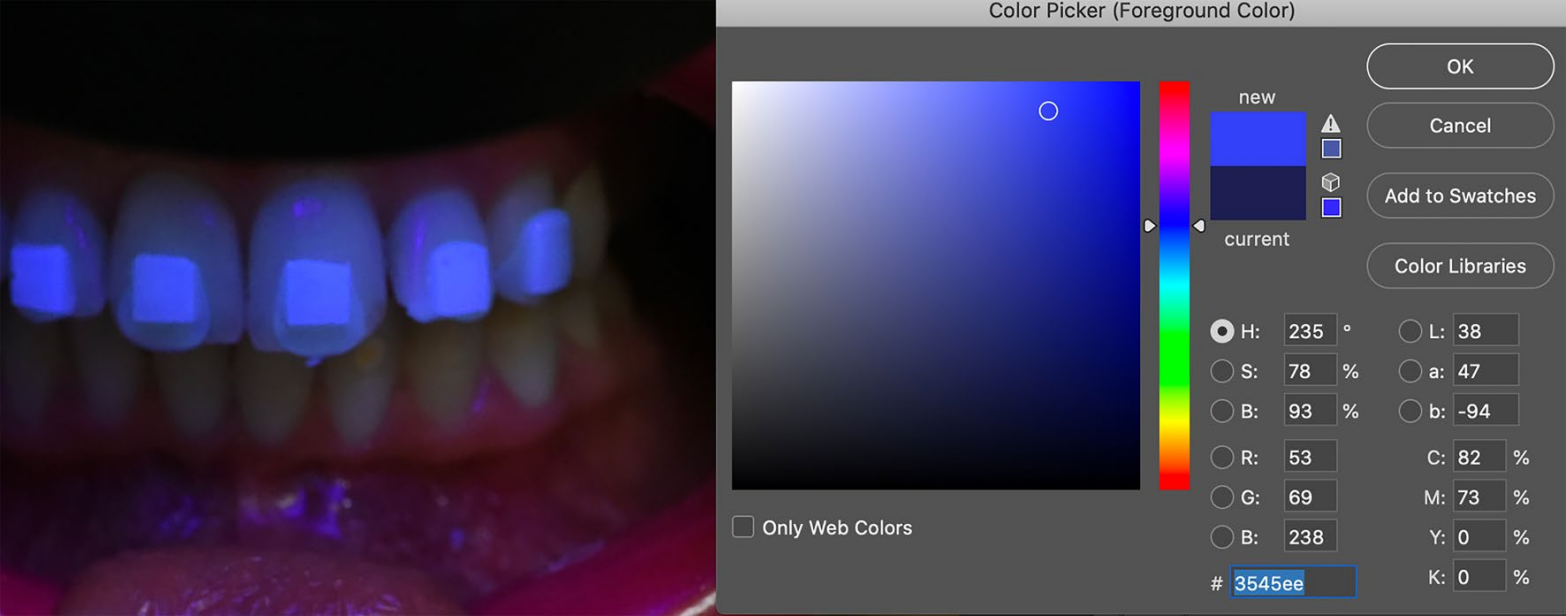
The “color picker” tool of the software to analyze the brightness differences between areas with and without adhesive

The “color picker” tool of the software (Adobe Photoshop, Version CC 2017; Adobe Incorporate, San Jose, CA, USA) was used to analyze the brightness differences between areas with and without adhesive (Fig. 3).

The brightness was picked up on the center of the adhesive area and on the center of the other half of the tooth.

The study design was reviewed and approved by the Ethics Committee of Postgraduate School of Orthodontics, Ferrara University, via Borsari 46, Ferrara, Italy (approval number 9/2019).

## Statistical analyses

Method error was assessed by means of repeated digitization of all measurements at a 14-day interval. No significant systematic errors were found between the measurement sessions. The method error turned out to be 0.2 (L* color coordinate).

A paired-sample t test was applied to compare the differences of the brightness values for each tooth. No statistically significant differences were found in the mean measurements between the different teeth and therefore the descriptive analysis was calculated including all teeth.

All of the statistical analyses were performed with the use of the same statistical software (Statplus Pro, version 6; AnalystSoft, Walnut, Calif).

## Results

**Fig. 4:**
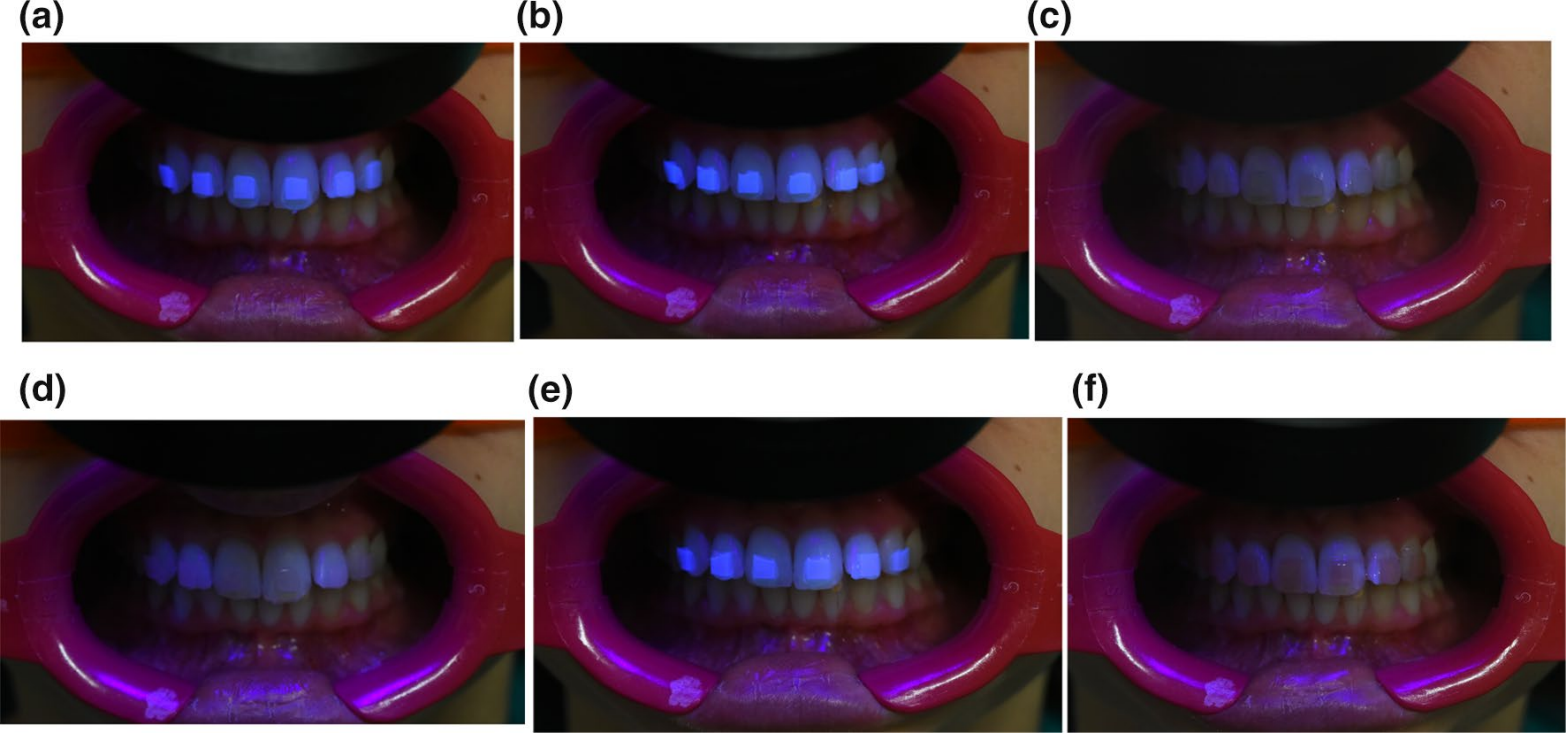
The pictures obtained from the camera to assess brightness; **a** GC Ortho Connect; **b** Gradia Direct LoFlo; **c** Greengloo; **d** Transbond XT; **e** KommonBase Clear; **f** KommonBase Pink

The pictures obtained from the camera were used to assess brightness values (Fig. 4a–f).

The “color picker” tool of the software allowed to register the brightness values of the areas with and without adhesive; furthermore, the average differences between the two different areas are reported in Table 1.

**Table 1:** Brightness values between the two different areas

	1.3 T	1.3 A	1.3 Δ	1.2 T	1.2 A	1.2 Δ	1.1 T	1.1 A	1.1 Δ	2.1 T	2.1 A	2.1 Δ	2.2 T	2.2 A	2.2 Δ	2.3 T	2.3 A	2.3 Δ	Avg Δ	Sd
Gc orthoconnect	14	33	19	22	38	16	25	42	17	34	47	13	35	49	14	24	38	14	15.5	2.3
Gradia LoFlo	17	38	21	23	43	20	26	45	19	35	49	14	41	53	12	31	43	12	16.3	4.1
Greengloo	12	15	3	19	17	− 2	21	21	0	31	28	− 3	34	31	− 3	25	27	2	− 0.5	2.6
Transbond XT	11	12	1	21	21	0	21	21	0	30	28	− 2	37	36	− 1	25	22	− 3	− 0.8	1.5
KommonBase pink	10	8	− 2	15	12	− 3	19	18	− 1	31	27	− 4	22	34	12	28	20	− 8	− 1.0	6.8
KommonBase clear	15	35	20	25	40	15	28	40	12	37	50	13	40	53	13	32	40	8	13.5	3.9

T, tooth brightness; A, adhesive brightness; Δ, difference of brightness between tooth and adhesive area; Avg Δ, average values of difference of brightness; Sd, standard deviation of brightness

GC Ortho Connect, Gradia Direct LoFlo and KommonBase Clear showed the highest differences of brightness (15.5, 16.3 and 13.5, respectively), while Greengloo, Transbond XT and KommonBase Pink registered similar values between adhesive area and tooth area with FIT (− 0.5, − 0.8 and − 1.0, respectively).

## Discussion

In the present study, the fluorescence behavior of 6 different orthodontic adhesives was tested with FIT. The amount of adhesive was standardized using the same template with a shell thickness of 1 mm for all analyzed resins. Furthermore, standardized conditions of taking photographs, UV LED flashlight and patient positioning were maintained throughout the procedure.

The brightness of adhesive area and tooth area (L* color coordinate) were measured on the photographs by using the “color picker” tool of the software. The same method with similar equipment has been used in a previous study, although 365 nm or 405 nm band-pass filters over the flashes and a green filter on the lens were employed for taking photographs [20]. One study concluded that the color coordinates values on photographs were highly correlated with the fluorescence intensities recorded by a spectrophotometer [22].

Fluorescent additives, as rare earths oxides (e.g., europium, cerium and ytterbium), are added to the glass fillers of resins in order to imitate the fluorescence properties of the dental substance; these additives allow fluorescence with FIT [6, 23, 24]. However, as previously reported, the precise composition of adhesive materials and especially the composition and concentration of the fluorescent substances are not declared by manufacturers [7, 21]. Furthermore, the amount of fluorescent properties for the orthodontic adhesives is still unknown [8, 17]. For all these reasons, this study can provide useful information for clinicians to use adhesives that can be easily removed at the end of orthodontic treatment.

The present study found that Gradia Direct LoFlo showed the highest brightness with FIT, followed by GC Ortho Connect and KommonBase Clear. These adhesives presented strong fluorescence properties and an easy detection during the bracket debonding with the FIT, while the other 3 adhesives showed low differences between areas with and without resin.

The results of this study are difficult to compare with previous ones in the literature owing to different methodologies employed.

This in vivo study simulated the real conditions with saliva and natural teeth in order to obtain reliable results; however, only frontal teeth were chosen to simplify the analysis.

Furthermore, only the fluorescence properties of restorative adhesive resin and of restorative CAD/CAM materials have been investigated [6, 7, 18].

Previous studies reported that the fluorescence properties could be independent from physical parameters of the material, as filler material, filler size distribution, filler shape, filler volume fraction or matrix resin material [6, 7].

The present study confirmed these outcomes, since no correlation was found between brightness and adhesive viscosity; in fact, the high viscosity adhesives resins, as Greengloo and Transbond XT, showed a similar performance in terms of fluorescence to the KommonBase Pink, the lowest viscous resin considered.

The results of this study underline the potential of using orthodontic adhesives with strong fluorescence properties in order to easily detect and remove the excess bonding remnants and to minimize possible enamel damages. FIT can simplify the orthodontic debonding procedures [13, 15–17, 25]. It is even more effective during the lingual brackets debonding, when distinguishing enamel and resins is even more complex for the less accessibility and for the greater interindividual morphological variability of lingual surfaces [16, 26].

During the orthodontic treatment, the use of auxiliaries should not damage the teeth and at the end of the treatment the same attention for the enamel is necessary [27, 28].

Engeler et al. reported that the FIT method was 100% successful and significantly superior to the non-FIT method and allowed the complete removal of all adhesive remnants after debonding of buccal and lingual brackets. The FIT method resulted in larger enamel defects on the lingual surfaces [29].

In addition, it has been reported that the adhesive remnant index (ARI) after lingual brackets debonding seems to be higher than the buccal one [30].

The objective of this study is to provide guidance to clinicians in choosing the proper adhesive.

The choice of a resin with higher fluorescence facilitates the removal of the orthodontic adhesive in the most appropriate way.

One of the major problems during debonding procedure is the adhesive remnants permanence or the dental enamel removal; therefore, fluorescence is one of the determining factors in the orthodontic adhesive choice.

This study has some limitations. Clinical conditions of orthodontic debonding have been researched; however, the etching and bonding procedures were avoided in order to facilitate the subsequent removal of the adhesive and to prevent alterations in enamel surface roughness.

## Conclusions

This in vivo study compared the fluorescent properties of 6 orthodontic adhesives; within the limitations of this current study, it was concluded that:

- The orthodontic adhesives showed different fluorescence properties.
- Some orthodontic adhesives are brighter with the FIT, facilitating identification and subsequent removal, while other orthodontic resins do not benefit from the FIT method.
- The viscosity of orthodontic adhesives does not influence the brightness emitted with FIT.

## Data Availability

Not applicable.

## Abbreviations

FIT: Fluorescence-aided identification technique
LED: Light-emitting diode
ARI: Adhesive remnant index
